# Lessons From Management: Perioperative Phacoemulsification Planning Following Resolution of Acute Angle Closure

**DOI:** 10.7759/cureus.14331

**Published:** 2021-04-06

**Authors:** Brendon W. H Lee, Fiona S Lau, Elizabeth L Wong, Danny Lam, Ian C Francis

**Affiliations:** 1 Faculty of Medicine, University of New South Wales, Sydney, AUS; 2 Ophthalmology, Prince of Wales Hospital, Sydney, AUS; 3 Ophthalmology, Sydney Hospital and Sydney Eye Hospital, Sydney, AUS; 4 Ophthalmology, Chatswood Eye Specialists, Sydney, AUS; 5 Ophthalmology, Chatswood Private Hospital, Sydney, AUS

**Keywords:** acute angle closure, phacoemulsification cataract surgery, glaucoma, zonular apparatus, perioperative planning, surgery, ophthalmology, eye, case report

## Abstract

Patients with loose zonular apparatus after acute angle closure may require phacoemulsification cataract surgery. The authors' experience from management of such patients provides excellent instruction on the surgical intervention for their cataracts. This is because patients who have recovered from acute angle closure glaucoma may not have evident zonular laxity preoperatively, as the iris may be taut secondary to the effects of associated ischaemia. If the surgeon’s preoperative planning is directed to the possibility of loose zonular apparatus, then appropriate preoperative, intraoperative, and postoperative planning and management can be effected. This may permit preoperative patient counselling regarding the potentially increased complexity of the case. Intraoperatively, deliberately gentle capsulorrhexis, the use of iris hooks or a pupil expander to dilate the pupil, iris hooks to support the capsular bag, and the employment of a capsular tension ring may be helpful. Postoperatively, due to the previous ocular ischaemia, intraocular pressure elevation may ensue, and should be actively managed. The authors provide a summary of factors that require consideration in patients undergoing cataract surgery following acute angle closure.

## Introduction

The zonular apparatus (ZA) is responsible for supporting the lens capsule of the eye. Loose ZA can lead to malposition or subluxation of the lens. Therefore, zonular laxity must be carefully evaluated in patients before undertaking phacoemulsification cataract and implant surgery (phaco). Loose ZA in patients who have recovered from acute angle closure (AAC) glaucoma represents an additional quandary, as such patients may not demonstrate zonular laxity on preoperative evaluation.

The authors present an illustrative case of a patient undergoing phaco with loose ZA secondary to AAC. A summary of factors that require consideration in patients undergoing phaco following AAC is provided. Further, the principles surrounding the preoperative, intraoperative, and postoperative planning and management of these factors in patients who have had AAC are highlighted.

## Case presentation

An 86-year-old lady with a medical history of hypertension, asthma, gout, and vitamin D deficiency presented with a three-day history of pain in the right eye and periorbital region. She had been recently noted by her referring optometrist to have developed blurred right vision associated with increasing nuclear sclerosis, manifested by a rapid myopic shift of -2.50 DS. There were no reported previous episodes of subacute angle closure.

Her cataracts were assessed as grade 3+ nuclear sclerosis in the right eye and grade 3 in the left (Lens Opacities Classifications System II) [1]. The right anterior chamber was flat temporally, demonstrating van Herick grade 0, while the left was van Herick grade 1 [2]. Gonioscopy confirmed a completely closed angle (Shaffer grade 0) in the right eye, with a grade 1-2 angle in the left eye [2].

Intraocular pressures (IOPs) were measured to be 45 mmHg in the right eye and 21 mmHg in the left eye using Goldmann applanation tonometry. The pupils reacted normally, but the right was slightly dilated (right 2.3 mm, left 1.7 mm). Following administration of oral acetazolamide and topical timolol/brimonidine (Combigan®), she underwent right corneal indentation for this episode of AAC [3,4]. After 10 cycles of corneal indentation, the patient’s right IOP settled to 19 mmHg.

Immediately following this IOP reduction, a single right YAG laser peripheral iridotomy was performed (7 x 2.6 mJ = 18 mJ). By the next day, the angle was noted to be more open (van Herick grade 1), and her IOP was 7 mmHg. Several small central Descemet’s membrane folds were noted. There was no evidence of iridodonesis. Her right preoperative corrected distance visual acuity (CDVA) was 6/18 part. On the same day, a prophylactic left YAG laser peripheral iridotomy was performed (8 x 2.6 mJ = 21 mJ).

In order to achieve definitive right anterior chamber stabilisation, to prevent future episodes of AAC, and to improve visual acuity, right phaco was recommended and performed.

During surgical capsulorrhexis, a tendency was noted for the capsular bag to displace nasally, inferring some temporal zonulolysis. Because of this, a Morcher® capsular tension ring (CTR) (Type 14, Stuttgart, Germany) was implanted immediately, and the surgery completed uneventfully. A small amount of residual cortex could not be aspirated because of its sequestration by the CTR.

On day 1 postoperatively, the patient had a right IOP of 17 mmHg, with a slightly nasally placed rhexis but a well-centred, hydrophobic acrylic, toric (1.5 DC) SN6AT3 intraocular lens (IOL) implant (Acrysof®, Alcon, Fort Worth, Texas). The patient’s day 1 postoperative CDVA was 6/6, but improved at one week to 6/4, with an IOP of 14 mmHg in each eye. Despite the patient’s slightly thick corneas (preoperative pachymetry of right 602 μm and left 601 μm), the visual outcome was satisfactory.

One month postoperatively, the patient’s IOP had elevated to 30 mmHg in the right eye and 27 mmHg in the left. The patient was using dexamethasone eye drops, but only in the right eye. Topical brimonidine (Alphagan®) was commenced bilaterally, and at five weeks postoperatively, the patient’s IOPs were 21 mmHg in each eye. Gonioscopy demonstrated angles of grade 1+ bilaterally. The right angle appeared slightly narrower than the left, most likely secondary to the patient’s pre-existing partially subluxated lens/capsular bag. The patient’s optic cup-to-disc ratios were 0.4 in the right eye and 0.35 in the left eye.

Twelve weeks later, uneventful left phaco surgery was performed, achieving a left CDVA of 6/4. At follow-up eight months later, CDVA was 6/4 part in each eye, with an IOP of 15 mmHg in each eye on no topical glaucoma medication. By 14 months postoperatively, the optic nerve cup-to-disc ratios were unchanged. Both eyes had also developed early posterior capsular opacification, and posterior capsulotomy with YAG laser was contemplated. 

## Discussion

The 86-year-old patient described achieved a satisfactory outcome in addressing her AAC, as well as achieving an acceptable visual outcome [5], with CDVA in each eye of 6/4 at one month, and 6/4 part at 14 months. While the right rhexis was noted to be slightly nasally displaced at day 1 postoperatively, it was central at 11 months postoperatively.

Although there was no preoperative iridodonesis, the capsulorrhexis did not behave normally intraoperatively, demonstrating laxity of the ZA. This directed the surgeon to the early placement of a CTR. This likely contributed to the satisfactory clinical outcome, both visually, in relation to her ZA laxity, and also in relation to her raised IOP.

To provide sound surgical perspective, the authors consider that the main indications for cataract surgery are those represented in Table 1. These are abbreviated as DIPVAX. This clearly includes patients with loose ZA after AAC.

**Table 1:** DIPVAX: The main indications for prioritised cataract surgery IOP, intraocular pressure

DIPVAX	
D	Dense cataracts and particularly high-grade nuclear sclerosis
I	Patients with Increasing dementia, aiming to improve visual function and general quality of life
P	Phacomorphic glaucoma
V	Diminished Visual acuity, especially in those patients with significant glare symptomatology, often confirmed by brightness acuity testing
A	Acute angle closure where laser iridotomy fails to open the angle adequately, or the risk of glaucomatous optic neuropathy remains high, including from poorly controlled IOP
X	PseudoeXfoliation with moderate, or worse, cataracts and poorly dilating pupils

However, given the difficulties anticipated and experienced in this case, care must be taken at every step to address potential complications. These include dehiscence of the ZA, anterior and posterior capsular tears in patients with more dense cataracts, and problems associated with small pupils. The latter may need to be addressed by the use of a pupil-dilating device, for example, a Malyugin ring. Further, because these cataracts are often dense and in brown eyes, capsular staining techniques are indicated. In this case, Vision Blue® (DORC International BV, Zuidland, Netherlands) was used. Iris hooks may also be used to achieve temporary intraoperative stabilisation of the capsular bag while the phaco is being done, and while IOL implantation is carried out.

A recent literature update by Fontana et al. also noted the important surgical point that even the first incision into the capsule by the capsulotomy needle may prove difficult in patients with ZA laxity [6]. Other strategies for dealing with ZA laxity during capsulorrhexis include the use of devices to provide counter-traction while the caspulorrhexis is being created. This can be accomplished by sequential placement of iris hooks placed in the partially created capsulorrhexis, use of a dialling hook placed in the capsulorrhexis, or use of a second microforceps to hold the capsulorrhexis. In all of these manoeuvres, adequate injection of dispersive viscoelastic assists the technique. While femtosecond laser-assisted technology cataract surgery could legitimately be considered as an appropriate capsulotomy technique for such patients, the surgeon might subsequently not know whether the ZA was defective until phaco had commenced [7]. In that event, a CTR may not already have been placed prophylactically, with potential untoward consequences. Similarly, in a large Malaysian study (n = 150,213), for example, pseudoexfoliation was positively associated with posterior capsular ruptures, and similar standard precautions need to be considered [8].

While a CTR was helpful in this case, had it been available, a Malyugin modified CTR may have been preferable, as suturing of the CTR to the sclera could have provided even greater IOL/capsular bag complex stability. While a satisfactory outcome was obtained in this case, there has been a recent report of chronic and intermittent angle closure caused by in-the-bag CTRs, and IOL dislocations in pseudoexfoliation cases [9]. This occurs in such cases because of anterior displacement of the capsular bag-CTR-IOL complex, and appeared resistant to treatment by laser peripheral iridotomy. Thus, capsular bag-CTR-IOL complex stability may be critical to long-term success; fortunately, it can be managed by capsular stabilising rings or segments.

At the World Glaucoma Congress in July 2017 held in Helsinki, Finland, the Effectiveness of Angle-closure Glaucoma of Lens Extraction (EAGLE) study demonstrated that cataract removal, while generally effective in minimising AAC, was not a risk-free procedure [10]. In other words, at every stage, following a previous episode of AAC, a high index of suspicion for potential surgical complications must be entertained. The surgical experience of the authors suggests both good surgical and visual outcomes following phaco surgery in the presence of previous phacomorphic glaucoma [11].

The authors consider that most ophthalmologists dealing with AAC would lower the IOP adequately enough to carry out a YAG laser iridotomy. They would then likely consider phaco surgery, especially if there were significant cataract present [11]. Significant intraoperative challenges may be generated by preoperative iridodonesis, unexplained shallowing or deepening of the anterior chamber, asymmetry of anterior chamber depth between the two eyes, unresolved raised IOP, and bound-down pupils. Corneas traumatised by high pressures, inflammation, and the ocular ischaemia of AAC may result in unexpected postoperative corneal decompensation, especially as the patient’s cornea may have sustained substantial endothelial cell loss.

Table 2 presents the key preoperative, intraoperative, and postoperative factors requiring management in patients undergoing phaco with previous AAC [10-16].

**Table 2:** Evaluation of 16 key preoperative, intraoperative, and postoperative factors in the management of cataracts in patients with previous acute angle closure

Sixteen key factors in the management of cataracts with previous acute angle closure	
Preoperative factors	1. Anterior chamber depth asymmetry
	2. Bound-down pupil
	3. Intraocular pressure variability
	4. Anterior chamber inflammation
	5. Maximum dilated pupil size
	6. Iridodonesis
	7. Nuclear sclerotic density
	8. Folds in Descemet’s membrane
	9. Presence of established glaucomatous optic neuropathy, including a relative afferent pupil defect
	10. Increased asymmetric pachymetric corneal thickness
Intraoperative factors	11. Difficult penetration of anterior capsule with capsulotomy needle
	12. Movement of entire lens on capsulorrhexis
	13. Movement of lens/capsular bag on attempted nuclear dialling following hydrodissection
	14. Unexpected lens movement during hydrodissection or phacoemulsification, if femtosecond laser-assisted technology cataract surgery is employed
Postoperative factors	15. Raised postoperative intraoperative pressure
	16. Persisting pachymetric corneal oedema

The EAGLE study reported in the Lancet in late 2016 demonstrated that clear lens extraction following AAC was superior to YAG laser peripheral iridotomy [10]. However, if the IOP is still raised despite maximum medical therapy of the AAC, iridotomy may not be possible due to impaired visualisation of the iris and marked anterior chamber shallowing. Further, there is the intrinsic risk of attendant corneal and iris burns. Hence, the authors recommend the implementation of effective corneal indentation [3,4]. Anecdotally, one patient seen in Myanmar by one of the authors (ICF) had had AAC for five days, with a pressure of 70 mmHg at presentation. A cataract of grade 4 NS was present, with marked corneal oedema. Corneal indentation was carried out for over 45 minutes, resulting in IOP normalisation, and resolution of the corneal oedema. This permitted an immediate YAG laser iridotomy, which was followed by uneventful cataract and implant surgery several days later.

## Conclusions

While the surgical and visual outcomes of phaco are generally satisfactory in eyes that have sustained AAC, the authors consider that addressing key perioperative factors as illustrated may facilitate the management of such patients.
